# Isolation of Endogenous TGF-β1 from Root Canals for Pulp Tissue Engineering: A Translational Study

**DOI:** 10.3390/biology11020227

**Published:** 2022-01-30

**Authors:** Matthias Widbiller, Andreas Rosendahl, Melanie Wölflick, Moritz Linnebank, Benedikt Welzenbach, Karl-Anton Hiller, Wolfgang Buchalla, Kerstin M. Galler

**Affiliations:** 1 Department of Conservative Dentistry and Periodontology, University Hospital Regensburg, 93053 Regensburg, Germany; andreas.rosendahl@ukr.de (A.R.); melanie.woelflick@ukr.de (M.W.); linnebank.moritz@gmail.com (M.L.); karl-anton.hiller@ukr.de (K.-A.H.); wolfgang.buchalla@ukr.de (W.B.); 2 Independent Researcher, 94034 Passau, Germany; b.welzenbach@gmx.de; 3 Department of Operative Dentistry and Periodontology, Friedrich-Alexander-University Erlangen-Nürnberg, 91054 Erlangen, Germany; kerstin.galler@uk-erlangen.de

**Keywords:** dental pulp, regeneration, tissue engineering, endodontics, cell homing, dentin matrix proteins

## Abstract

**Simple Summary:**

Tissue engineering of the dental pulp has been a goal of dental research for years. In this translational study, a chairside protocol is designed using endogenous dentin matrix proteins as signaling molecules for pulp regeneration. These bioactive molecules can be isolated from root canals by ultrasonic-activated irrigation, further processed chairside, and mixed with a hydrogel. The scaffold material is to be injected into the root canal and effect cell homing, i.e., allowing stem cells from the periapical space to migrate into the root canal. The aim of this innovative approach is the formation of an innervated and vascularized connective tissue that resembles the pulp in form and function.

**Abstract:**

Cell homing for dental pulp tissue engineering has been advocated as a feasible approach to regenerate dental pulp in a clinical setting. In order to develop a translational protocol for clinical application, we wanted to determine the effects of disinfectants on the availability of growth factors from the root canal, the amount that can be obtained in this context, and whether they can be processed for use in tissue engineering procedures. The extraction of growth factors should also be confirmed in a clinical setting. Root canals were prepared in 36 extracted mature teeth, and the amount of TGF-β1 in solution was quantified after different irrigation protocols (sodium hypochlorite, chlorhexidine) and after intracanal medication (calcium hydroxide). Centrifugal filters with a cut-off of 10,000 Da and 3000 Da were used for efficient concentration, and volumes and amounts of retained TGF-β1 were measured at different time points. During conventional endodontic treatment, ethylenediaminotetraacetic acid (EDTA) solution was collected after ultrasonic activation from the root canals of mature teeth of 38 patients, and growth factor content was quantified via enzyme-linked immunosorbent assay (ELISA). Irrigation with sodium hypochlorite reduced TGF-β1 release into EDTA. This effect was partially reversed by canal enlargement after the use of sodium hypochlorite and by subsequent use of calcium hydroxide. A few minutes of centrifugation with a cut-off of 10,000 Da reduced the initial volume of the irrigant by 90% and led to a continuous increase in concentration to the same extent. Furthermore, TGF-β1 was obtained from root canals of mature teeth during endodontic treatment in quantities that have been shown to elicit desirable cellular responses in a subsequent clinical application. A mixture with a suitable scaffold material and injection into the root canal has the potential to promote dental pulp regeneration.

## 1. Introduction

Recent clinical studies demonstrate impressively that dental pulp regeneration after transplantation of pulpal stem cells into an empty root canal in cases of irreversible pulpitis or pulp necrosis is possible [1,2]. A translation of such cell-based approaches into dental practice is hampered by strict regulations and high operational costs. Therefore, cell homing has been advocated as a potential strategy and feasible alternative [3,4]. Cell homing does not rely on cell transplantation but aims at the recruitment of local stem cells into a custom-made scaffold material laden with bioactive molecules that are inserted into the defect or site of injury [5,6]. The presence of mesenchymal stem cells in the apical region has been demonstrated, along with their potential to differentiate into pulp- and dentin-forming cells [7]. Different injectable scaffold materials have been tested for pulp regeneration, where fibrin-based materials are looked upon favorably [8,9,10]. The dentin matrix has been discovered as a reservoir for various bioactive molecules, which are easily accessible and can be released during root canal treatment with conventionally used irrigation solutions such as EDTA [11,12]. The EDTA-soluble fraction of the dentin matrix has been studied extensively, and effects on pulpal stem cell differentiation and tertiary dentin formation have been proven [13,14]. A recent proteomic analysis of the dentin matrix revealed a total amount of over 800 proteins, which exhibit high diversity in regard to their functions [15]. In the context of tissue engineering and regeneration, these proteins include chemotactic, angiogenic, and neurogenic factors, cytokines, as well as multiple growths and differentiation factors [13,16]. As these proteins are released during tooth development and become embedded and bound to the dentin matrix, they are unique to this tissue and intimately associated with odontoblast differentiation and formation of the dentin–pulp complex. Exposure of dentin to EDTA, a chelator and demineralizing agent, which is commonly used during conventional root canal treatment for smear layer removal, results in the release of these proteins from the dentin matrix [14,17]. Growth factors in EDTA have been quantified, and the number of growth factors can be increased after ultrasonic activation of the irrigant [18]. Proteins from the dentin matrix exert chemotactic effects on dental pulp stem cells and promote cell differentiation towards an odontoblast-phenotype [14,19].

**Figure 1:**
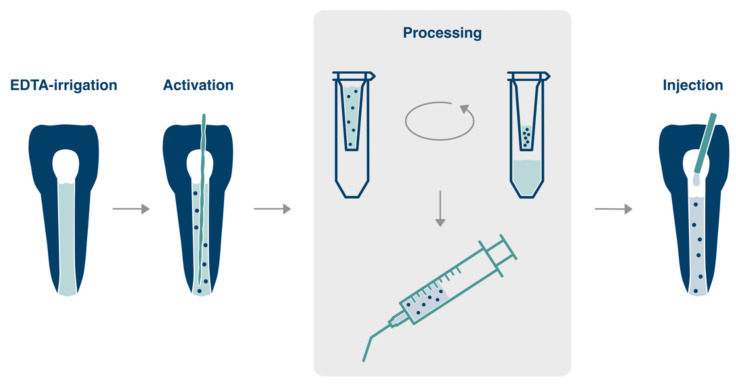
Envisioned clinical procedure. After root canal preparation and disinfection, EDTA is used to remove the smear layer. After refreshment of EDTA, the solution is activated by ultrasound, which releases growth and differentiation factors from the dentin surface. The solution is removed from the canal, and proteins are concentrated via a chairside centrifugation step. A hydrogel biomaterial, e.g., a fibrin-based scaffold, is mixed with concentrated dentin matrix proteins and injected back into the root canal in contact with the periapical tissues.

A combination of a fibrin-based scaffold material with dentin-derived growth factors harvested during root canal preparation and irrigation and the re-insertion of this mixture into the root canal after cleaning and disinfection might be a feasible approach to recruit stem cells from the periapical tissues into the canal [3]. These cells can migrate, populate the scaffold, degrade and replace it with the extracellular matrix, proliferate and differentiate and thus fill the formerly empty root canal with new pulpal tissue. Specific details of the envisioned clinical concept are visualized in Figure 1.

While the primary indication for regenerative endodontic procedures is currently seen in immature teeth, the concept is also conceivable in mature teeth. As initial studies on blood-clot-based techniques show, biological approaches are promising in adults and, therefore, could take on an even broader indication in the dental treatment spectrum in the future [20,21].

New tissue formation in empty root canals was investigated in vivo in a cell homing model recently [22]. Root canals of extracted human teeth were prepared and filled with a fibrin-based material enriched with dentin matrix proteins. A cell pellet of dental pulp stem cells was placed at the root tip, and the constructs were implanted subcutaneously in the backs of immunodeficient mice. After four weeks, tissue ingrowth was observed, which was significantly higher in groups with dentin matrix proteins compared to control specimens without. Furthermore, cell differentiation along the existing root canal wall was evident, along with new dentin matrix formation.

Overall, previous work has already laid the foundations for the protein composition of the dentin matrix, the optimal selection of irrigants, and the specific release dynamics in the root canal. In addition, minute amounts of dentin matrix proteins have been found to induce chemotactic effects as well as differentiation and mineralization [14]. However, these findings need to be brought together, aligned, and complemented to some extent in order to establish a clinical protocol. In addition, answers to the following application-related questions must be found:

1. How do disinfecting agents affect the amounts of growth factors, and can the irrigation and disinfection protocol be adapted to a subsequent regenerative treatment?
2. Is it possible to process growth factors in a chairside protocol after collection from root dentin such that these can be mixed with a biomaterial and injected back into the canal at a sufficiently high concentration?
3. Is it feasible to collect sufficient amounts of growth factors during conventional root canal treatment?

Therefore, the aim of this study was to develop a chairside protocol for the collection of growth factors in sufficient quantity from root dentin during root canal treatment in preparation for re-insertion into the canal in combination with a fibrin-based biomaterial. In this context, cytotoxic EDTA needs to be eliminated or neutralized, and importantly, handling as well as time aspects that are critical factors of a clinical protocol have to be considered.

## 2. Materials and Methods

### 2.1. Optimization of Release Parameters Ex Vivo

In order to address the first two questions as stated in the introduction, a standardized ex vivo model was established to investigate the impact of individual steps of the clinical procedure during root canal treatment on the release of growth factors from root canal wall dentin. Therefore, 36 single-rooted, caries-free and unrestored teeth were collected after extraction and stored until further use (0.5% Chloramin T trihydrate, Merck, Darmstadt, Germany). Access cavities were created, a manual glide path was prepared with Kerr Files ISO 10 to 25 (VDW, Munich, Germany) to working length (1 mm short of root length), and all teeth were allocated equally to three groups according to respective working lengths. Samples were fixed in polystyrene containers (Sterilin™ 7 mL polystyrene bijou containers, Thermo Fisher Scientific, Waltham, MA, USA) with impression material (Silaplast Futur, DETAX, Ettlingen, Germany), followed by standardized root canal preparation with RECIPROC^®^ blue R25 to size 25, 0.08 taper (VDW, Munich, Germany) under continuous irrigation with 0.9% saline (Sodium chloride solution, Sigma-Aldrich, St. Louis, MO, USA).

#### 2.1.1. Step 1: Irrigation

In order to investigate the influence of the disinfecting irrigant on subsequent growth factor release by EDTA, conventional needle irrigation with 20 mL for 5 min was carried out in three groups as follows:

1. 2% sodium hypochlorite (Hypochlorit-SPEIKO^®^, Dr. Speier, Bielefeld, Germany)
2. 0.2% chlorhexidine (Chlorhexidine digluconate solution, Sigma-Aldrich, St. Louis, MO, USA)
3. 0.9% saline (control)

Root canals were dried with paper points, rinsed with 20 mL of 10% EDTA disodium salt dihydrate (EDTA-Na_2_, AppliChem, Darmstadt, Germany) for 5 min to remove the smear layer and neutralize the previous irrigant, and dried again. Root canals were again filled with EDTA, which was activated for 20 s with an ultrasonic file (IRRI K 25/25, VDW, Munich, Germany) and the appendant unit (VDW.ULTRA^®^, VDW, Munich, Germany) in irrigation mode. Subsequently, the solution was collected with a syringe, and the procedure was repeated until a total volume of 100 µL was obtained (first sampling), the number of repeats was documented. Samples were immediately frozen at −80 °C and stored until further use.

#### 2.1.2. Step 2: Root Canal Enlargement

Irrigants not only act on the surface but also penetrate deep into the dentin tubules. To verify whether the release of growth factors from root dentin can be improved by removing the affected superficial layer before exposure to EDTA, a second preparatory step was carried out. Root canals were enlarged further with RECIPROC^®^ blue R50 to size 50, 0.05 taper (VDW, Munich, Germany) while saline was used for irrigation. After drying with paper points, EDTA was used, activated as before, and 100 µL of solution was collected, frozen, and stored (second sampling).

#### 2.1.3. Step 3: Intracanal Dressing with Calcium Hydroxide

Root canals were rinsed with saline, dried, and calcium hydroxide paste (UltraCal^®^ XS, Ultradent Products, South Jordan, UT, USA) was injected. Teeth were incubated (100% humidity, 37 °C and, 5% CO_2_) for 72 h. Calcium hydroxide was removed by irrigation with EDTA, and canals were dried. In analogy to the previous samplings, EDTA was injected, activated as described above, and 100 µL of solution was collected (third sampling).

The concentration of TGF-β1 was quantified for each sample by a solid-phase sandwich ELISA for TGF-β1 (Human TGF-beta 1 Quantikine ELISA Kit, R&D Systems™, Wiesbaden, Germany), which was chosen as a representative growth factor within the mixture of dentin matrix-derived proteins. A previously established approach with a double set of standards was used to accurately calculate growth factor concentrations [3]. Based on volume and concentration, the mass of TGF-β1 collected from each root canal was calculated. Data from 12 samples per group (n = 12) were analyzed to compute median values and 25–75% percentiles. Furthermore, the obtainable volume per tooth and the number of collections that were necessary to reach 100 µL were determined for all teeth, and median values were calculated (n = 36).

### 2.2. Concentration and Preparation Ex Vivo

In order to develop a chairside procedure for concentration of growth factors harvested from root dentin, centrifugal filters with a molecular weight cut-off of 3000 Da and 10,000 Da (Amicon^®^ Ultra—0.5 mL Ultracel^®^—3 K/10 K, Merck, Darmstadt, Germany) were tested for their efficacy to retain EDTA-soluble growth factors and to reduce the volume of the chelating agent to a minimum. Dentin matrix proteins were extracted from human molars as described previously [14], and a solution with a final concentration of 100 pg/mL of TGF-β1 in phosphate-buffered saline (PBS without Ca^2+^, Mg^2+^, Biochrom GmbH, Berlin, Germany) was prepared. The centrifugal filters were loaded with 500 µL of solution and centrifuged in a cooling centrifuge (Rotina 420, Andreas Hettich, Tuttlingen, Germany) at 21 °C and 14,000× *g*. Concentrated samples were recovered from the filter devices via reverse spin technique after 3, 6, 9, 12, 15, and 18 min. For both filter versions and each time point, volumes of the collected samples were determined, and TGF-β1 was quantified as described before (n = 6). Medians with 25–75% percentiles were calculated, and a four-parameter logistic regression (4PL) was generated to illustrate the time course of volume decrease and increase in TGF-β1-concentration, respectively (GraphPad Prism 9; GraphPad Software, La Jolla, CA, USA).

### 2.3. Cytotoxic Effects of EDTA and Neutralization

In order to determine the minimum cytotoxic concentration of EDTA on local cells in the envisioned clinical protocol, cell culture experiments with stem cells from the apical papilla (SCAP) were carried out. Different concentrations and forms of EDTA (EDTA-Na_2_, EDTA-CaNa_2_) and citric acid (pH 1.7, pH 6) were added in culture, and cell viability was assessed at different time points. In addition, neutralization of EDTA by addition of CaCl_2_ and thus elimination of toxic effects was investigated. Methodical details for the cell culture studies can be found in the Supplemental File.

### 2.4. In Vivo Availability of TGF-β1

In order to evaluate the availability of dentin-derived proteins from patients in a clinical setting, EDTA was collected from root canals during routine endodontic treatment. The procedure, as well as the collection of data, was in accordance with ethical guidelines of the University of Regensburg. After a pilot phase, in which the protocol was adapted, and handling aspects were optimized, 38 teeth scheduled for routine endodontic treatment were included with informed consent of the patients. After isolation of the respective tooth with rubber dam and the preparation of access cavity and glide path, working length was determined electronically. Root canal preparation to 35, 0.04 under copious irrigation with 2% sodium hypochlorite (Hypochlorit-SPEIKO^®^, Dr. Speier, Bielefeld, Germany) was carried out, intracanal dressing (UltraCal^®^ XS, Ultradent Products, South Jordan, UT, USA) was placed, and the tooth was sealed temporarily. It was assured that the intracanal dressing remained for at least two weeks and not longer than four weeks. In a second visit, the dressing was removed by irrigation with 2% sodium hypochlorite, and canals were dried and filled with EDTA, which was activated for 30 s with an ultrasonic file as described above for smear layer removal. EDTA was refreshed and left inside the canal for 10 min, then removed with a carpule syringe and sterile cartridge (Aesculap, Tuttlingen, Germany) and collected. Canals were dried with sterile paper points, and 0.9% saline was injected, activated as described above for 30 s, removed and collected. Samples were stored frozen at −80 °C until further analysis by ELISA as described. Various case-dependent parameters were documented, among them tooth type (anteriors, premolars, molars), clinical situation (irreversible pulpitis, pulp necrosis, retreatment), patient age (<40, 40 to 60, >60), and extent of coronal restoration (none, ≤50%, >50% area of access cavity). Data were obtained, and medians with 25–75% percentiles were calculated.

### 2.5. Statistical Analysis

For all ex vivo and in vitro experiments, data were treated non-parametrically (Shapiro–Wilk) and analyzed in pairs using the Mann–Whitney *U*-test on an α = 0.05 level of significance (GraphPad Prism 9; GraphPad Software, La Jolla, CA, USA). *p*-values were adjusted familywise for multiple comparisons by the error rates method, and statistically significant differences between groups were indicated by asterisks. In analogy, in vivo results were processed by SPSS (version 25.0, SPSS Inc., Chicago, IL, USA). However, after professional mathematical advice, statistical analysis was limited to the total results, and case-dependent parameters were not considered due to the uneven distribution of data within the groups.

## 3. Results

### 3.1. Release Parameters Ex Vivo

**Figure 2:**
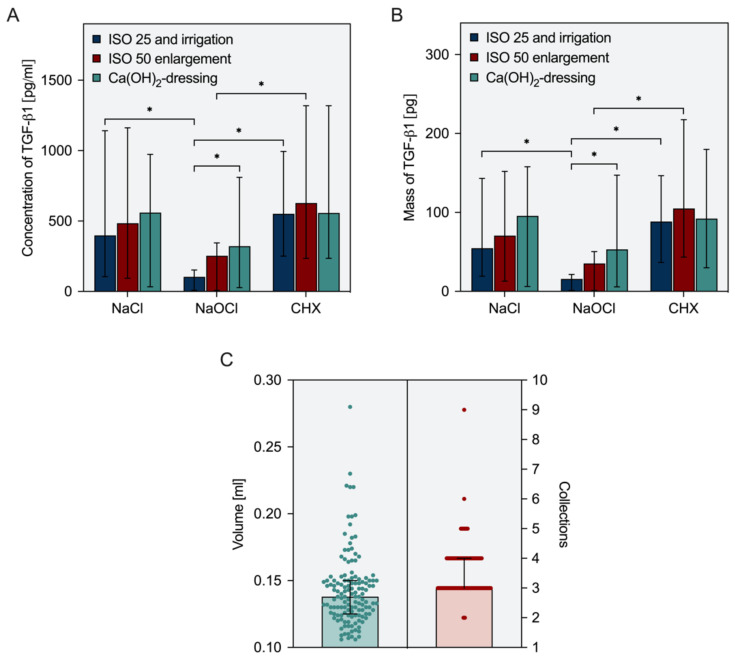
Collection of growth factors from root canals in an ex vivo model. Release of TGF-β1 depending on irrigant solution after a 3-step-protocol including initial preparation, root canal enlargement, and the use of an intracanal dressing. (**A**) Concentration and (**B**) absolute mass of soluble TGF-β1 in EDTA collected from root canals after ultrasonic activation (n = 12). (**C**) Retrieved volumes of EDTA and number of collections necessary to obtain a minimum total volume of 100 µL (n = 36). Depicted are median values and 25–75% percentiles (column and error bars) as well as single measurements (points). Width of distribution of points proportionate to the number of points. Asterisks indicate statistical differences between groups.

The findings on growth factor release by EDTA after the use of different disinfectants and the volumes obtained after each collection step are summarized in Figure 2A,B. Irrigation with sodium hypochlorite reduced the release of TGF-β1 from root dentin significantly compared to controls with saline (*p* ≤ 0.0160), but also compared to chlorhexidine (*p* ≤ 0.0160), which did not exhibit any negative effects but rather enabled the highest release rates. An enlargement of the root canals from size 25, 0.08 to 50, 0.05 resulted in slightly higher amounts of TGF-β1 in all groups without significant differences compared to the measurements following step 1 (*p* > 0.0719). After the use of sodium hypochlorite for irrigation, the combination of canal enlargement and subsequent use of calcium hydroxide facilitated a significant increase in concentrations and masses of TGF-β1 (*p* ≤ 0.0397), thus partially rescuing the deleterious effects of sodium hypochlorite on growth factor release from root canal wall dentin. In teeth irrigated with saline or chlorhexidine, these additional measures had only negligible effects (*p* > 0.4796). Across all groups, a median total volume of 138 µL was collected from the root canals. To achieve this volume, three or four collection steps were sufficient in 90% of all cases (Figure 2C).

### 3.2. Concentration and Preparation Ex Vivo

**Figure 3:**
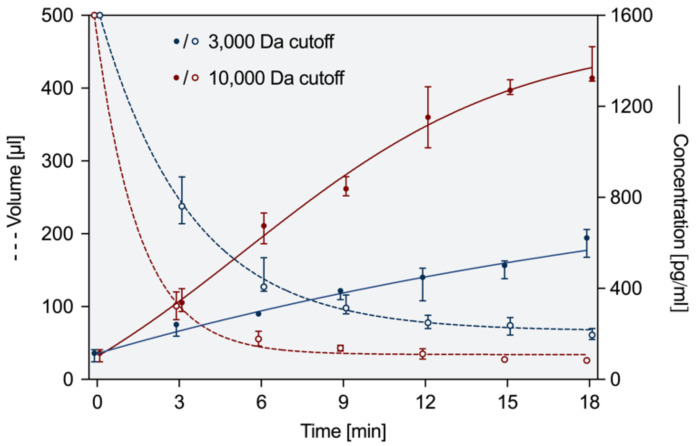
Volume of EDTA and concentration of TGF-β1 after centrifugation in filters with a cut-off at 3000 or 10,000 Da. Both parameters were measured at 6 time points (n = 6). Depicted are medians and 25–75% percentiles.

At the endpoint after 18 min of centrifugation, the initial volumes of 500 µL were reduced to 61 µL with a 3000 Da-cutoff and to 26 µL with a 10,000 Da-cutoff (Figure 3), whereas the volume in 3000 Da centrifugal filters was decreased by half within three minutes, this result was achieved twice as fast with 10,000 Da filters after 1.5 min. During centrifugation, the concentration of TGF-β1 increased from 100 pg/mL to 622 pg/mL with 3000 Da filters and to 1324 pg/mL with 10,000 Da filters. The differences observed for the two different filters were statistically significant both for volume and concentration at 3 min and later (*p* ≤ 0.0022).

### 3.3. Cytotoxic Effects of EDTA and Neutralization

In cell culture, EDTA-Na_2_ was cytotoxic in a concentration-dependent manner. Even 0.1% EDTA (5.26 mM) reduced the viability of SCAP to 65% after 24 h. No toxic effects were evident at higher dilutions. Calcium chloride (CaCl_2_) by itself was not toxic, and an addition of 2.68 mM of CaCl_2_ completely abolished the toxic effects of 5.26 mM EDTA. The respective figures can be found in the Supplemental File.

### 3.4. In Vivo Availability of TGF-β1

**Figure 4:**
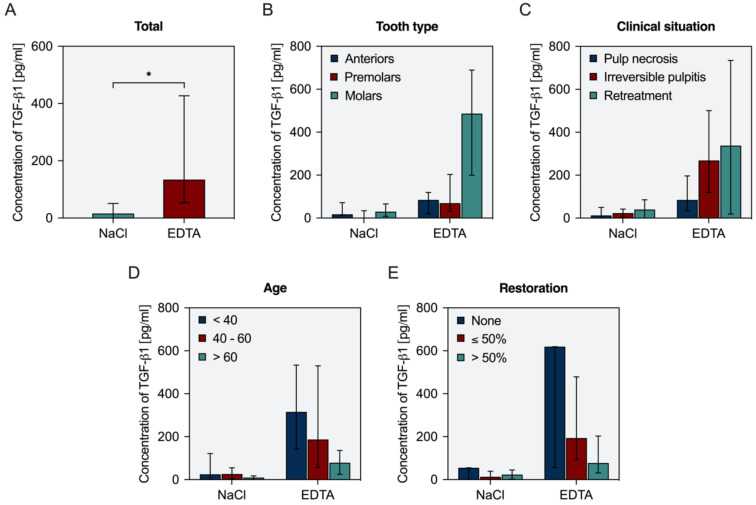
Release of TGF-β1 into EDTA and saline for all 38 teeth (**A**) and according to tooth type (**B**), clinical situation (**C**), patient age (**D**) and extent of the coronal restoration (**E**). Depicted are median values with 25–75% percentiles. Asterisks indicate statistical differences between groups.

The amounts of growth factor collected during endodontic treatment were significantly higher (*p* < 0.0001) in EDTA than in NaCl (Figure 4A). However, there were considerable variations with individual patients. A more detailed analysis with regard to potentially influencing factors revealed that the available amount of TGF-β1 was higher in molars compared to anteriors and premolars (Figure 4B) and also higher in vital and retreatment cases compared to necrotic cases (Figure 4C). With increasing age, the number of growth factors released by EDTA decreased steadily (Figure 4D). An increasing extent of the coronal restoration also affected the amount of TGF-β1 released by EDTA (Figure 4E).

## 4. Discussion

The idea of regenerating dental pulp rather than filling the void with a synthetic material in cases of pulp necrosis has raised considerable interest in the past years. Clinical procedures for regenerative endodontic treatment have been developed, where provocation of bleeding into the root canal can promote new tissue formation to replace the lost pulp [23,24]. Evidence exists to demonstrate tooth survival and healing of periapical lesions in immature teeth, along with a potential for root lengthening and thickening, which may reduce the risk of root fractures in these compromised teeth [25,26,27]. In analogy to immature teeth, clinical studies report similar results in terms of survival and healing after applying the same principle to mature teeth [20,21]. Furthermore, platelet-rich plasma and platelet-rich fibrin have also shown promising results in regenerative endodontic applications with regard to the outcome parameters described above [28,29]. Despite the fact that these procedures induce the formation of ectopic tissue inside the root canal rather than restoring dental pulp in its original structure and function, the development of these clinical protocols has contributed considerably towards the goal of a “biological root canal filling” [3,5]. 

While these clinical applications have become part of the endodontic treatment spectrum, tissue engineering-based therapies have been developed in a laboratory as well as in vivo studies. However, clinical evaluations are scarce [1,2,30]. A primarily cell-free approach following the principle of cell homing appears to be rather promising in the short-term development of regenerative strategies, as it requires only a modification of the current protocols for conventional root canal treatment. The current study aimed to overcome some of the last hurdles of making cell homing a clinically applicable endodontic procedure.

The envisioned protocol includes, after preparation and disinfection of the root canal, the use and ultrasonic activation of EDTA to release growth factors from the dentin surface of the root canal walls. Furthermore, it contains the collection of this solution, concentration by centrifugation, and subsequent mixture with a fibrin-based biomaterial that will be injected into the canal. After coronal restoration, the chemotactic factors within the mixture of dentin-derived proteins could attract resident stem cells into the root canal, which can populate the space, degrade the biomaterial, differentiate and form new pulpal tissue.

### 4.1. Release Parameters Ex Vivo

A first issue that arises is that the use of disinfectants and other chemicals may interfere with the release of bioactive proteins from the dentin matrix [31]. It is known that sodium hypochlorite reduces the number of proteins due to its proteolytic effects, which are based on chlorination and oxidation. This may result in a significant decrease in available bioactive proteins. While disinfection is paramount during endodontic treatment, alterations to the protocol have to be considered, which suffice both the claims of disinfection and sufficient growth factor release. The design of the ex vivo study presented here was chosen to enable the investigation of various parameters, namely the use of irrigation solutions (sodium hypochlorite and chlorhexidine), the instrumentation of canals, and the use of an intracanal medicament (calcium hydroxide). The findings from the respective experiments suggest that sodium hypochlorite has detrimental effects on subsequent growth factor release, whereas saline, chlorhexidine, and calcium hydroxide do not. Enlargement of the canal by use of saline after the first phase of preparation under sodium hypochlorite disinfection increased growth factor release, but this effect is not significant. As disinfection and tissue dissolution during endodontic treatment is essential, one might consider a modified protocol of preparation and irrigation such that sodium hypochlorite is only used during the first phase of preparation. Final enlargement of the canal may be carried out with either saline or chlorhexidine as an irrigation solution. Furthermore, after disinfection, calcium hydroxide is preferred as an intracanal dressing because, on the one hand, it does not harm the periapical cells [32] and, on the other hand, it generally does not affect the release of growth factors [31]. Especially after the application of sodium hypochlorite, which penetrates the tubules and degrades organic matter such as dentin matrix proteins even after the root canal has dried [33], calcium hydroxide seems to have an attenuating function due to its less tissue-dissolving properties. During the second visit, only EDTA is used as an irrigant, similarly to the recommended protocol for regenerative endodontic procedures [23,24]. The use of chlorhexidine as an endodontic irrigant is discussed controversially. While it does not dissolve organic compounds and may elicit unfavorable changes in biofilm structure, 2% chlorhexidine in liquid form had a similar microbial performance against several microorganisms as 5.25% sodium hypochlorite, and bacterial growth was inhibited after a contact time of only 15 s [34,35,36]. In addition to disinfecting and preserving endogenous growth factors, maintaining stem cell viability and promoting their proliferation and differentiation are also critical for clinical success. An optimized protocol for chemical disinfection is important to control the conditions that influence the fate of stem cells prior to their delivery into the canal [32]. At clinically relevant concentrations, chlorhexidine may, directly and indirectly, affect SCAP survival, which can be overcome by limiting the irrigation time and by neutralization with L-α-lecithin [37]. Therefore, chlorhexidine may, in principle, represent a disinfectant that is favorable both for cells and growth factors during regenerative procedures.

### 4.2. Concentration and Neutralization

Previous in vitro studies reported chemotactic effects of dentin matrix proteins at concentrations as low as 10 pg/mL TGF-β1, cell differentiation and mineralization showed an optimum at 500 pg/mL [14]. Ex vivo experiments have shown that it is possible to obtain EDTA-soluble proteins from root canals in amounts that can exert biological effects within a clinically acceptable period of time. 

Since centrifuges are regularly used in dental practices (e.g., for platelet concentrates in oral surgery or periodontal surgery), it also seems practicable to further process the dentin-derived proteins with the help of centrifuge filters. In this context, a cut-off of 10,000 Da is appropriate as it retains the sought-after proteins, e.g., TGF-β1 with a molecular mass of 25 kDa, and at the same time allows for a sufficient reduction in volume in a reasonable time span [15]. In the course of centrifugation, the efficiency of the concentration decreased slightly over time, and early on, the concentration of growth factors reached a sufficient level. As the patient is waiting in a treatment situation during the centrifugation process, it seems reasonable to weigh up the duration and quantity and to choose an early time point as an end point, e.g., 6 min. 

Whereas the toxicity of EDTA is of concern, it will be reduced in two ways: by dilution and by the addition of calcium (chloride). In the solution used in this study (EDTA-Na_2_), EDTA molecules are already binding sodium ions. However, the agent is further capable of chelating divalent cations, e.g., Ca^2+^. Once chelation of calcium has occurred (EDTA-CaNa_2_), cytotoxic effects are no longer evident. Thus, calcium released from root canal dentin by EDTA will contribute to its neutralization. Furthermore, residual EDTA will be saturated by calcium chloride, which is an essential component present in larger quantities in fibrin-based scaffold materials [38].

### 4.3. In Vivo Availability of TGF-β1

The clinical data show that a release of growth factors is possible in an endodontic treatment setting. Interestingly, not only inter-individual variations can be observed, but also patient age, clinical situation, type of tooth, and extent of the coronal restoration may be influencing factors. In the case of retreatment, higher amounts of growth factors may be explained by the increased surface area of dentin due to repeated and enhanced canal preparation. Furthermore, retreatment was mostly performed in molars. With regards to pulp necrosis, previous studies have shown that the metabolically active bacteria in the intracanal biofilm are able to break down peptides by proteolytic enzymes such as collagenases, metalloproteases, serine proteases, and extracellular peptidases [39]. Thus, the destruction of bioactive proteins of the extracellular dentin matrix by infection-related enzymes is conceivable. Furthermore, molars show larger contact areas as they exhibit three or more canals and commonly present with larger access cavities compared to incisors and premolars. Similarly, the extent of the coronal restoration influences growth factor release. These findings lead to the plausible explanation that the size of the surface area of dentin that comes into contact with EDTA correlates with the amount of growth factor that can be collected. In aged patients, the surface area may shrink due to more extensive restorations as well as occluded areas within the root canal system and sclerosis of dentinal tubules [40]. A critical point may be the high variability of growth factor release among patients. However, even minute concentrations of growth factors are biologically effective, as described above. As it is not clear how this will affect clinical outcomes and whether, in addition to the release, the response rate also varies, this point needs to be evaluated in further in vivo studies. 

The differences in the yield of growth factors ex vivo and in vivo are, of course, due to the clinical variations described as well as to the fact that, for better standardization, only single-rooted teeth without restorations were used in the ex vivo experiments. Moreover, the standardized instrumentation in the ex vivo model differed significantly from the root canal preparation in vivo, which was adapted to the circumstances of each individual treatment case. While the ex vivo situation was easier to control, the removal of the irrigation in vivo was more difficult due to the accessibility of the tooth and the treatment situation. Furthermore, it must be acknowledged that the clinical trial was initiated at the same time as further optimization of the protocol was investigated ex vivo. The original approach was to obtain growth factors by activation of saline to circumvent EDTA-cytotoxicity. Thus, EDTA was not activated in the clinical setting but left in place for 10 min in accordance with previous studies on growth factor release [31]. However, it has been shown in the meantime that ultrasonic activation can enhance the effects of EDTA, where acoustic streaming of the liquid in a narrow space can increase growth factor release significantly [18]. A collection of growth factors in saline as a non-toxic agent did not prove successful. This fact and the concurrent advances in the dilution and neutralization of EDTA led to the adaptation of the concept to reproducibly obtain a high amount of growth factors from root canals.

Based on the evidence available in the literature and the results presented in this study, it is possible to develop a clinical protocol for cell homing with dentin-derived proteins. However, this approach must be investigated and evaluated in-depth in further in vivo studies before a clinical application can be envisaged. A detailed protocol would include the following steps over two treatment sessions:

First visit:
  1. Initiation of root canal preparation under copious irrigation with sodium hypochlorite at 2%,
  2. further canal enlargement and use of saline (or chlorhexidine),
  3. intracanal dressing with calcium hydroxide and temporary seal (2 to 4 weeks).
Second visit:
  4. Removal of calcium hydroxide by irrigation with EDTA,
  5. refreshment of EDTA inside the canal and ultrasonic activation for 30 s,
  6. collection of EDTA solution from the canal and repetition until 100 µL of solution are available (3 to 4 times),
  7. transfer of the solution to a filter with a molecular weight cut-off of 10,000 Da, addition of ultrapure water to a volume of 500 µL (1:4 dilution), spin for 6 min,
  8. mix with a fibrin-based biomaterial,
  9. re-insert into the root canal in contact with the periapical tissues,
  10. cover the material, e.g., with a hydraulic calcium silicate cement, and place an adhesive seal.

## 5. Conclusions

Generally, it seems clinically feasible to isolate dentin matrix proteins during the endodontic treatment of patients, to prepare them accordingly, and finally to add them to a scaffold material in the course of a cell-homing approach. The findings presented here form the basis for further in vivo studies and allow the planning of a pilot study with the described protocol, preferably on teeth with simple root canal anatomy, such as single-rooted teeth, which could bring us one step closer to the goal of pulp regeneration.

## Data Availability

The data presented in this study are available on request from the corresponding author.

## Supplementary Materials

The following supporting information can be downloaded at: https://www.mdpi.com/article/10.3390/biology11020227/s1, Materials and Methods/Results: Cytotoxicity of Irrigants; Neutralization.
