# Supplementary material for: Isolation of Endogenous TGF-β1 from Root Canals for Pulp Tissue Engineering: A Translational Study
Source: Biology (Basel). 2022 Jan 30;11(2):227. doi: 10.3390/biology11020227 (PMC8869556; doi:10.3390/biology11020227)
Supplement: Supplementary file 1 [file biology-11-00227-s001.zip › biology-1541966-supplementary.pdf]

Supplemental File

Cytotoxicity of Irrigants

Materials and Methods

Cells were obtained from the apical papilla of freshly extracted third molars with informed consent of the patients and approval by the Ethics Committee (Faculty of Medicine, University of Regensburg, Regensburg, Germany). The isolation and characterization as mesenchymal stem cells were carried out according to an established protocol [1]. Stem cells of the apical papilla (SCAP) were cultured in αMEM with 10 % fetal bovine serum (FBS), 50 µg/mL L-ascorbic acid 2-phosphate, 100 U/mL penicillin and 100 µg/mL streptomycin at 37 °C with 5 % CO_2. All reagents for cell culture were purchased from Gibco™ (Thermo Fisher Scientific, Waltham, MA, USA).

SCAP of passage 5 were seeded into 96-well plates at a concentration of 4,000 cells/well to investigate cytotoxicity of decalcifying irrigants. Culture media were supplemented by disodium EDTA (EDTA-Na_2; Ethylenediaminotetraacetic acid disodium salt dihydrate, AppliChem, Darmstadt, Germany), calcium disodium EDTA (EDTA-$CaNa_2$; Ethylenediaminetetraacetic acid calcium disodium salt, Merck, Darmstadt, Germany) and citric acid at pH 1.7 and pH 6 (Citric acid monohydrate, Merck, Darmstadt, Germany) in final concentrations of 0.6 %, 0.45 %, 0.3 %, 0.15 %, 0.1 % and 0 % (control). After 24 h, cell viability was determined by methylthiazolyldiphenyl-tetrazolium bromide (MTT) assay. Cells in each well were incubated with 100 µL of 0.5 mg/mL MTT solution (Thiazolyl Blue Tetrazolium Bromide; Sigma-Aldrich, St. Louis, MO, USA) for 60 min at 37 °C and 5 % CO_2. Subsequently, the dye was dissolved in 200 µL/well of dimethyl sulfoxide (DMSO; Thermo Fisher Scientific, Waltham, MA, USA) at 250 rpm shaking for 20 min. Optical density readings were performed on a microplate reader at $\lambda = 540$ nm (Infinite® 200; Tecan, Männedorf, Switzerland). Medians and 25 - 75 % percentiles were calculated on basis of repeated experiments and normalized to the respective control (n = 8).

Results

Citric acid at pH 1.7 and EDTA-Na_2 affected viability of SCAP after 24 h significantly ($P \leq 0.0011$). The effect was concentration dependent and even 0.1 % citric acid (pH 1.7) and EDTA-Na_2 reduced cell viability to 77 % and 65 % respectively. However, citric acid at pH 6 hardly exerted negative effects and EDTA-$CaNa_2$ even lead to a slight increase of viability.

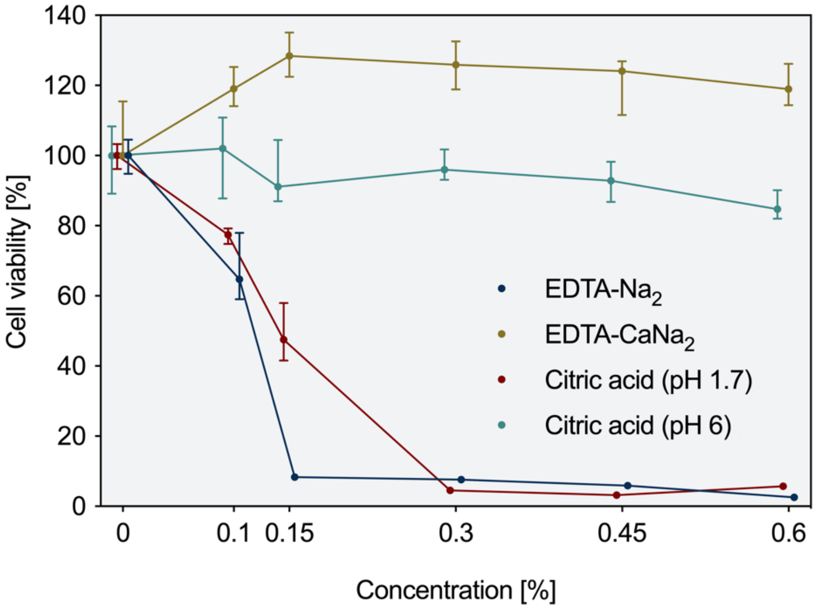

Neutralization

Materials and Methods

Additionally, the possibility to attenuate the effects of EDTA-Na_2 by addition of $CaCl_2$ was investigated. Therefore, SCAP were seeded as described before and exposed to 5.26 mM EDTA-Na_2 (0.1 % in medium) with $CaCl_2$ in a final molarity of 2.68 mM, 5.26 mM, 10.52 mM or 21.04 mM (Calcium chloride dihydrate, Carl Roth, Karlsruhe, Germany). MTT assays were performed after 24 h and results from repeated experiments were summarized as medians with 25 - 75 % percentiles (n = 16).

Results

In the neutralization experiment, EDTA-Na_2 again reduced SCAP-viability ($P < 0.0001$) whereas increasing concentrations of $CaCl_2$ alone were not detrimental. The toxicity of EDTA-Na_2 was neutralized by addition of $CaCl_2$ from a concentration of 2.68 mM ($P < 0.0001$) and even higher amounts did not show significant differences from the group without EDTA-Na_2 ($P > 0.5705$).

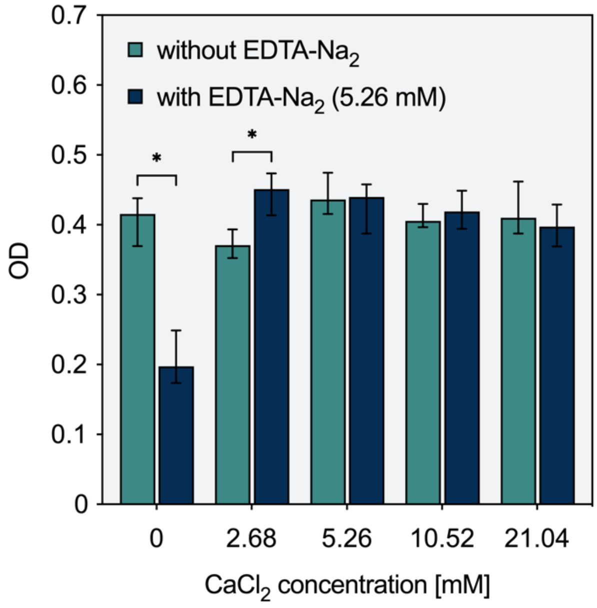

References

1. Widbiller, M.; Lindner, S.R.; Buchalla, W.; Eidt, A.; Hiller, K.-A.; Schmalz, G.; Galler, K.M. Three-dimensional culture of dental pulp stem cells in direct contact to tricalcium silicate cements. *Clinical Oral Investigations* **2016**, 20, 237–246.
